# Oral health status of students with visual or hearing impairments in Northeast China

**DOI:** 10.1186/s12903-023-02923-1

**Published:** 2023-04-26

**Authors:** Jian Li, Kaiqiang Zhang, Chang Cha, Zhenfu Lu, Lu Liu

**Affiliations:** Department of Preventive Dentistry, School and Hospital of Stomatology, Liaoning Provincial Key laboratory of Oral Diseases, China Medical University, Nanjing North Street, No.117, 110101 Shenyang, China

**Keywords:** Visual impairment, Hearing impairment, Dental, Oral hygiene

## Abstract

**Background:**

Visual or hearing impairments in students seriously affect their quality of life. The aim of this study was to identify oral hygiene status and its influencing factors on visual or hearing impairments in students in Northeast China.

**Methods:**

This study was conducted in May 2022. A total of 118 visually impaired students and 56 hearing impaired students from Northeast China were included in this study via census. Oral examinations and questionnaire-based surveys of students and their teachers were conducted. The oral examinations included caries experience, prevalence of gingival bleeding and dental calculus. The questionnaires included three parts: Social demographics (residence, sex and race) and parents’ educational level; Oral hygiene habits and medical treatment behaviors; Knowledge and attitudes towards oral health care. This questionnaire was selected from the Fourth China National Oral Health Survey and the reliability and validity of the questionnaire were previously tested. T tests, one-way ANOVA, *χ*^2^ tests and multivariate logistic analyses were conducted to evaluate the differences and dependent variables of dental caries.

**Results:**

The prevalence of dental caries in visually impaired and hearing impaired students were 66.10% and 66.07%. The mean number of DMFT, prevalence of gingival bleeding and dental calculus in visually impaired students were 2.71 ± 3.06, 52.08% and 59.38%, respectively. The mean number of DMFT, prevalence of gingival bleeding and dental calculus in hearing impaired students were 2.57 ± 2.83, 17.86% and 42.86%, respectively. The results of the multivariate logistic analysis showed that fluoride use and parents’ educational background had an impact on the caries experience of visually impaired students. The daily toothbrushing frequency and parents’ educational background had an impact on the caries experience of hearing impaired students.

**Conclusions:**

The oral health situation of students with visual or hearing impairments remains severe. It is still necessary to promote oral and general health in this population.

## Background

Oral health is a common disease that affects people’s quality of life. According to a World Health Organization (WHO) report, oral health has become one of the diseases affecting the quality of life of hundreds of millions of people [1]. The WHO recently estimated that the world’s blind population is 285 million, of which 19 million are children [2]. Most of the current studies that were aimed at studying oral health in individuals with visual impairments revealed that compared with that of adolescents without visual impairments, the oral health of visually impaired teenagers is poorer [3–6], although some studies showed that the oral health of visually impaired teenagers is better than that of normal adolescents [7].

Worldwide, almost 360 million people suffer from hearing impairment (5.3% of the world’s population) [8]. Because hearing impaired patients cannot receive oral health education and cannot easily communicate with dental personnel, the risk of oral diseases increases [9]. Therefore, a large proportion of people with disabilities only seek emergency care instead of preventive or restorative care.

The oral health problems of hearing impaired and visually impaired individuals have attracted attention. Visually impaired children face difficulties in maintaining oral health. When brushing their teeth, they cannot see visible caries or gingival bleeding, which affects their oral health and early dental examinations [10]. Due to communication difficulties with listening and speaking, hearing impaired individuals are unable to receive timely and effective treatment.

As a developing country with the largest population, China has a large number of people with disabilities. There are 2.46 million school-age children with disabilities aged 6–14 years, accounting for 2.96% of the total disabled population, of which 130,000 are visually impaired children and 110,000 are hearing impaired children. Most of them receive education in ordinary or special schools [11]. At present, there are few studies on the oral health problems of visually impaired and hearing impaired individuals. Meanwhile, there is a lack of relevant epidemiological survey data for these populations in China.

The present study investigated the oral health status and associated factors of the students who attended the only school for the visually impaired and the only school for the hearing impaired in Northeast China. In this study, a census method was used to select students from the only school for the visually impaired and the only school for the hearing impaired in Northeast China.

## Methods

### Subjects

The sample of this study was composed of two groups: the visually impaired students were recruited from the only independent visual impaired school in Shenyang and the hearing impaired students were recruited from the only independent hearing impaired school in Shenyang. The sampling method was census. The inclusion criteria were students who were diagnosed with visual or hearing impairments and whose parents or guardians signed informed consent forms. The exclusion criteria were students who were unable or unwilling to complete the oral examination. The 118 visually impaired students (71 males and 47 females), ranging in age from 6 to 31 years old and the 56 hearing impaired students (29 males and 27 females), ranging in age from 6 to 21 years old, attended this survey. According to previous reports [11], visual impairment and hearing impairment were divided into 4 grades, of which grades 1 and 2 were severe and grades 3 and 4 were mild. In addition to oral examinations of the people in these two special schools, we also conducted a questionnaire survey for their parents/guardians and the school teachers. Oral examinations and questionnaires were administered after written informed consent was obtained from the parents and teachers. This study was approved by the Ethics Committee of the School of Stomatology, China Medical University (K2022048).

### Data collection

The oral clinical examination was carried out under an artificial light source using a CPI probe. The components of the examination included decayed–missing–filled teeth (DMFT), pit and fissure sealing, gingival bleeding and dental calculus. Dental caries, gingival bleeding and dental calculus were diagnosed according to WHO criteria [12]. The inspection was conducted in strict accordance with the standards of the Fourth National Oral Health Survey. All oral examinations in this study were completed by two highly qualified dentists who had passed theoretical and practical training for the Fourth National Oral Health Survey in China. In addition to the examiner, to ensure the reliability of the data, a standard examiner was established, and 5% of the examiners were randomly checked to compare the data. The reliability of the data was determined by the Kappa value between the two examiners. The Kappa value for the dental caries-related examinations was required to be higher than 0.8, and the Kappa value for the periodontal-related examinations was required to be higher than 0.6.

The questionnaire interviewers who underwent national screening, training and certification collected the questionnaire information from the parents and teachers in an efficient and unbiased manner. The coincidence rate of the questionnaire answers between each interviewer and trainer were required to exceed 95%. Then, the questionnaire interviewers went to the school classroom and explained the purpose and matters needing attention to the parents. The main contents of the questionnaire included three parts. The first part was social demographics (residence, sex and race) and parents’ educational level (low: junior school or below, median: senior school, high: college or above). The second part was oral hygiene habits and medical treatment behaviors: (1) frequency of tooth brushing ( ≧ once / day, once / day, < once / day or never); (2) brushing teeth by themselves (by themselves, partially need others help, completely need others help); (3) the brushing methods (horizontal brushing, vertical brushing, rotating brushing, no regular method, never brush); (4) the use of fluoride (none, once/year, twice/year); (5) frequency of sugar consumption habits including sweet drinks, cakes and candies ( ≧ twice / day, once / day, < once / day or never); (6) medical treatment behaviors: frequency of dental visits in the last year (once, twice, three times, never), etc.; The third part was knowledge and attitudes towards oral health care: (1) There is no need to treat deciduous teeth caries (agree, disagree, unknown); (2) Fluoride is useless to dental protection (agree, disagree, unknown); (3) Pit and fissure sealant can prevent dental caries of children (agree, disagree, unknown); (4) Teeth are born good or bad, no correlation with the protection (agree, disagree, unknown); (5) It is important to do oral examination regularly (agree, disagree, unknown). The questionnaire were selected from the Fourth China National Oral Health Survey [13]. The reliability and validity of the questionnaire were previously tested. And for each item, the correlation coefficient with other items within the factor was greater than those of the other factors. These analyses confirmed the validity of the questionnaire. After the questionnaire was completed, the questionnaire was further checked by a specially assigned person in charge, and incomplete questionnaires were returned and continued until the questionnaires were completed. All the students who participated in the oral examination had finished the questionnaire. Therefore, there was no missing data in this study.

### Data analysis

A statistical software package (IBM SPSS Statistics, V.20) was used for data analysis. Caries prevalence (%), the mean DMFT (decayed, missing and filled teeth) score, the prevalence of gingival bleeding and dental calculus were calculated. The statistical significance (mean DMFT score) of differences in caries experiences was assessed by an independent sample t test (two categories) and a one-way ANOVA (more than two categories). *χ*^2^ tests were used to compare proportions. Multivariate logistic analyses were performed to investigate the influence of the independent variables on the students’ dental caries experiences. The statistical significance level of all tests was set at 0.05.

## Results

### General characteristics

In this study, 118 visually impaired students (average age 15.8) and 56 hearing impaired students (average age 13.3) in Northeast China participated in this survey. A total of 94.06% percent of the students had grade 1 or 2 visual impairment (referred to as blind), and 92.85% had grade 1 or 2 hearing impairment (referred to as deaf). There were 28 students aged 6–12 years and 90 students aged 13–30 years with visual impairment and 7 students aged 6–12 years and 49 students aged 13–30 years with hearing impairment. The distributions of all the relevant factors are presented in Table 1.

**Table 1:** Overall status of dental caries in visually impaired and hearing impaired students

School	Variables	Group	N(%)	Pit and fissure sealant	DT	MT	FT	DMFT	Caries prevalence rate
Visual impairment	Gender	Male	71(60.17%)	2(1.69%)	1.86 ± 2.51	0.03 ± 0.17	0.44 ± 1.05	2.34 ± 2.85	63.38%
		Female	47(39.83%)	4(3.39%)	2.83 ± 3.21	0.02 ± 0.16	0.43 ± 1.25	3.28 ± 3.31	70.21%
	Age	6–12	28(23.73%)	2(1.69%)	3.32 ± 3.79	0	0.54 ± 1.37	3.86 ± 3.99	64.29%
		13–30	90(76.27%)	4(3.39%)	1.91 ± 2.40	0.03 ± 0.18	0.40 ± 1.05	2.36 ± 2.64	66.67%
	Level	Low	7(5.93%)	0	2.17 ± 1.83	0	0.17 ± 0.41	2.33 ± 1.86	66.67%
		Serious	111(94.07%)	6(5.08%)	2.27 ± 2.90	0.03 ± 0.16	0.45 ± 1.16	2.76 ± 3.12	66.67%
	Total		118	6(5.08%)	2.25 ± 2.84	0.03 ± 0.16	0.43 ± 1.13	2.71 ± 3.06	66.10%
Hearing impairment	Gender	Male	29(51.79%)	5(8.93%)	2.41 ± 2.98	0.03 ± 0.19	0.24 ± 0.95	2.69 ± 2.98	65.52%
		Female	27(48.21%)	2(3.57%)	1.44 ± 1.67	0.56 ± 2.01	0.44 ± 1.42	2.44 ± 2.71	66.67%
	Age	6–12	7(12.50%)	2(3.57%)	2.86 ± 3.85	0	0.29 ± 0.78	3.14 ± 3.93	57.14%
		13–21	49(87.50%)	5(8.93%)	1.82 ± 2.23	0.33 ± 1.51	0.35 ± 1.25	2.49 ± 2.68	67.35%
	Level	Low	4(7.14%)	0(0%)	2.50 ± 3.11	1.75 ± 3.50	0	4.25 ± 4.43	75.00%
		Serious	52(92.86%)	7(12.50%)	1.90 ± 2.44	0.17 ± 1.12	0.37 ± 1.24	2.44 ± 2.69	65.38%
	Total		56	7(12.50%)	1.95 ± 2.47	0.29 ± 1.41	0.34 ± 1.20	2.57 ± 2.83	66.07%

### Caries experiences of the studied students

The prevalence and severity of dental caries in the participating students are summarized in Table 1. The prevalence of dental caries in visually impaired and hearing impaired students were 66.10% and 66.07%. The mean DMFT score of visually impaired students was 2.71 ± 3.06. The pit and fissure sealing rate was 5.08%. The mean DMFT score of hearing impaired students was 2.57 ± 2.83. The pit and fissure sealing rate was 12.50%. There were no significant differences in sex, age, or impairment degree among the DMTF, MT and FT variables between the two schools.

All the included students had at least one first permanent molar eruption. The overall prevalence of caries in the first molar was 52.54%, and the mean number of caries of the first molar was 1.22 ± 1.42 in visually impaired students. The overall prevalence of caries in the first molar was 50.00%, and the mean number of caries of the first molar was 1.21 ± 1.50 in hearing impaired students. There was no significant difference in sex, age, or impairment degree level between the two schools (Table 2).

**Table 2:** Caries of the first permanent molar and use of pit and fissure sealant

School	Variables	Gender		Age (years)		Level		Total
		M	F	6–12	13–30	Low	Serious	
Visual impairment	Numbers of students	71(60.17%)	47(39.83%)	28(23.73%)	90(76.27%)	7(5.93%)	111(94.07%)	118
	Number of first permanent molar	280(60.46%)	183(39.52%)	103(22.25%)	360(77.75%)	24(5.18%)	439(94.81%)	463
	Number of students with caries of the first permanent molar	35(56.45%)	27(43.55%)	11(17.74%)	51(82.26%)	3(4.84%)	59(95.16%)	62
	Prevalence of caries of the first permanent molar (%)	49.30%	57.45%	39.29%	56.67%	42.86%	53.15%	52.54%
	Number of caries of the first permanent molar	84(57.93%)	61(42.07%)	30(20.69%)	115(79.31%)	6(4.14%)	139(95.86%)	145
	Mean number of caries of the first permanent molar	1.08 ± 1.36	1.43 ± 1.50	1.04 ± 1.50	1.28 ± 1.40	1.00 ± 1.10	1.24 ± 1.44	1.22 ± 1.42
Hearing impairment	Numbers of students	29(51.79%)	27(48.21%)	7(12.50%)	49(87.50%)	4(7.14%)	52(92.86%)	56
	Number of first permanent molar	116(51.79%)	108(48.21%)	28(12.50%)	196(87.50%)	16(7.14%)	208(92.86%)	224
	Number of students with caries of the first permanent molar	16(57.14%)	12(42.86%)	4(14.29%)	24(85.71%)	2(7.14%)	26(92.86%)	28
	Prevalence of caries of the first permanent molar (%)	55.17%	44.44%	57.14%	48.98%	50.00%	50.00%	50.00%
	Number of caries of the first permanent molar	36(53.73%)	31(46.27%)	10(14.92%)	57(85.07%)	6(8.96%)	61(91.04%)	67
	Mean number of caries of the first permanent molar	1.31 ± 1.47	1.11 ± 1.55	1.29 ± 1.38	1.20 ± 1.52	1.50 ± 1.91	1.19 ± 1.48	1.21 ± 1.50

### Prevalence of gingival bleeding and dental calculus in the studied students

As shown in Table 3, the prevalence of gingival bleeding in the participants with visual impairment was 52.08%, and the prevalence in males was significantly higher than that in females (*P* < 0.05). The prevalence of gingival bleeding in students with hearing impairment was 17.86%, which was lower than that in students with visual impairment. The prevalence of dental calculus was 59.38% and 42.86% in students with visual impairment and those with hearing impairment, respectively.

**Table 3:** Prevalence of gingival bleeding and dental calculus

School	Variables		N	Number of students with gingival bleeding	%	*χ2*	*P* value	Number of students with dental calculus	%
Visual impairment	Gender	Male	59	36	61.02% *	4.90	0.027	34	57.63%
		Female	37	14	37.84%			23	62.16%
	Age (years)	6–12	7	1	14.29%	4.32	0.038	3	42.86%
		13–30	89	49	55.06%			54	60.67%
	Status	Low	5	4	80.00%			5	100.00%
		Serious	90	46	51.11%			53	58.89%
	Total		96	50	52.08%			57	59.38%
Hearing impairment	Gender	Male	29	7	24.14%			15	51.72%
		Female	27	3	11.11%			9	33.33%
	Age (years)	6–12	7	1	14.29%			1	14.29%
		13–30	49	9	18.37%			23	46.94%
	Status	Low	52	9	17.31%			23	44.23%
		Serious	4	1	25.00%			1	25.00%
	Total		56	10	17.86%			24	42.86%

### Results of the questionnaires and oral health knowledge

The response and completion rates of the survey were 100%. Caries experience (mean DMFT score) based on the influencing factors is shown in Table 4. The mean DMFT score of students who used fluoride and brushed their teeth more than once per day was lower than that of students who did not (*P* < 0.05). Caries experience also showed that students with parents with a high educational background had a lower mean DMFT score (*P* < 0.05). Among those with visual impairment, students who brushed their teeth by themselves had a lower DMFT score than the students who completely or partially needed help from others. Among students with hearing impairment, students who brushed their teeth more than once per day and had a higher parental education background had a lower DMFT score (*P* < 0.05).

**Table 4:** Caries prevalence in different family factors with DMFT scores as dependent variable respectively

School	Variables		N (%)	Dental Caries		
				DMFT (SD)	t / F	*P* value
Visual impairment	Fluoride use	None	37(31.36%)	3.65 ± 3.78	4.76	
		Once/ year	42(35.59%)	2.93 ± 2.93 ^#^		0.045
		Twice /year	39(33.05%)	1.59 ± 1.98 *		0.003
	Frequency of brushing/ day	≥ Once / day	40(33.90%)	2.33 ± 2.75	3.81	
		Once / day	62(52.54%)	2.47 ± 2.53		
		<Once / day or Never	16(13.56%)	4.63 ± 4.80*		0.011
	Brush teeth by themselves	By themselves	80(67.80%)	2.26 ± 2.65	3.78	
		Partially need others help	33(27.97%)	3.39 ± 3.45		
		Completely need others help	5(4.24%)	5.40 ± 4.83*		0.025
	Parent’s education background	low (junior school or below)	40(33.90%)	3.40 ± 3.65	4.90	
		median (senior school)	63(53.39%)	2.78 ± 2.78		
		high (college or above)	15(12.71%)	0.99 ± 0.25*		0.002
Hearing impairment	Frequency of brushing/ day	≥ Once / day	17(30.36%)	2.00 ± 2.37	6.59	
		Once / day	18(32.14%)	1.28 ± 1.60		
		<Once / day or Never	21(37.50%)	4.14 ± 3.31*		0.014
	Parent’s education background	low (junior school or below)	14(25.00%)	3.50 ± 3.88	3.11	
		median (senior school)	20(35.71%)	3.15 ± 2.03		
		high (college or above)	22(39.29%)	1.46 ± 2.39*		0.033

DMFT：decayed–missing–filled teeth

**Fig. 1:**
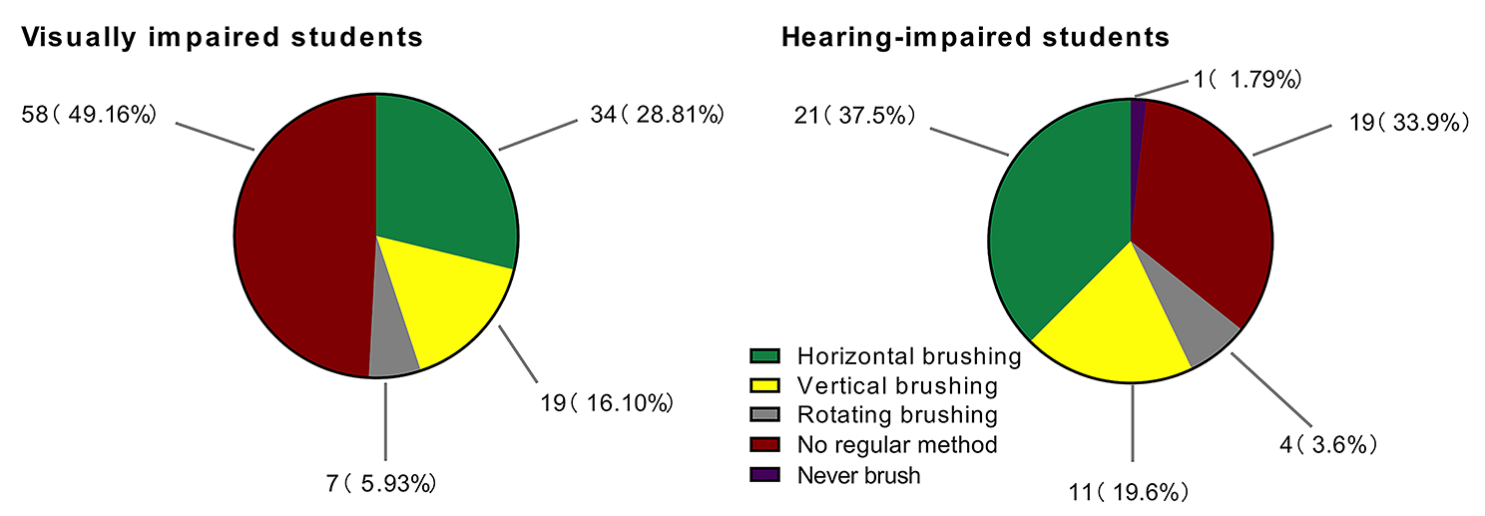
Brushing ways of students

**Fig. 2:**
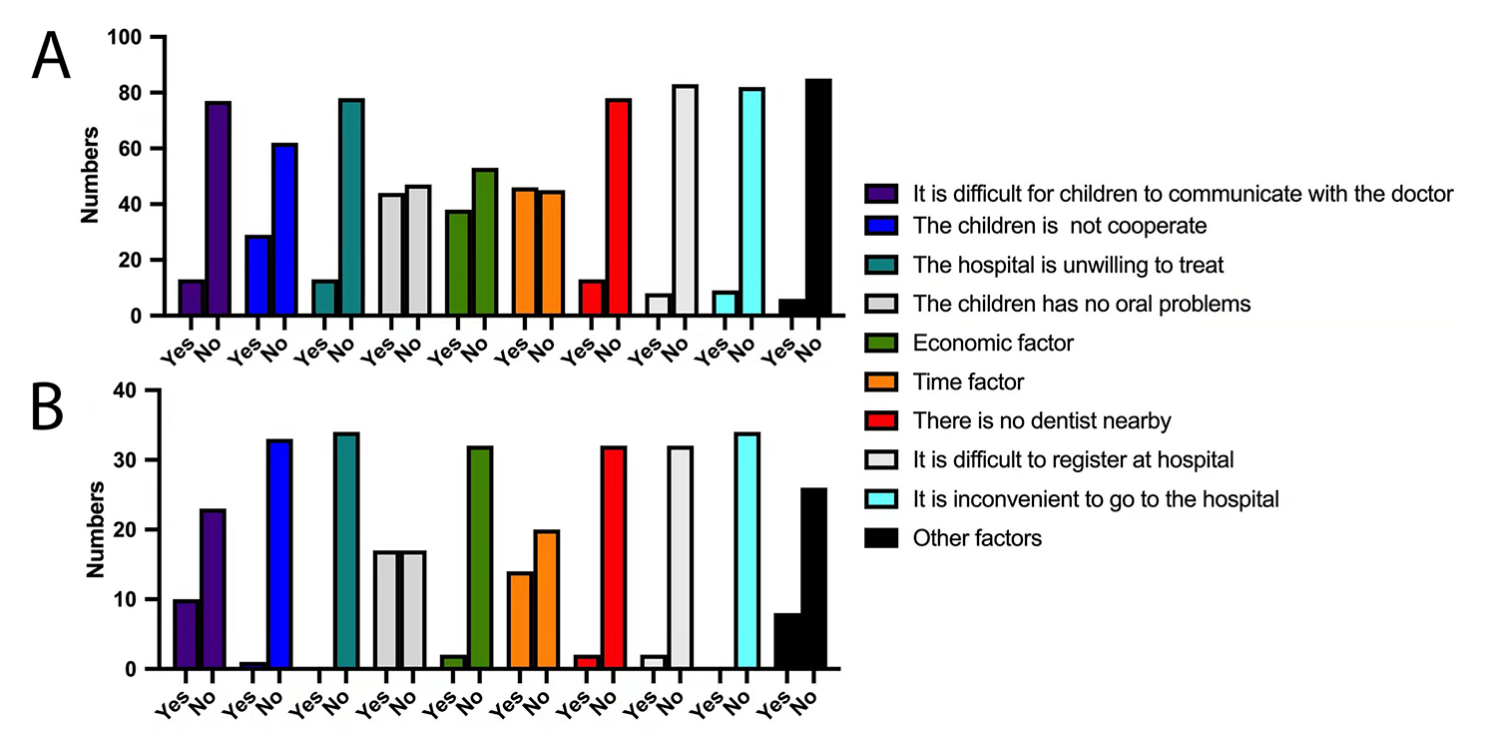
The reasons why students did not go to the dental institution

We further analysed the brushing methods of these students. As shown in Figs. 1 and 28.81% of the visually impaired students used the horizontal brushing method, 16.10% used the vertical brushing method, 5.93% used the rotating brushing method and 49.16% did not have a fixed brushing method. The results for hearing impaired students are shown in Figs. 1 and 37.50% of the hearing impaired students used the horizontal brushing method, 19.64% used the vertical brushing method, 7.14% used the rotating brushing method and 33.93% had no fixed brushing method, 1.79% never brush teeth. As shown in Fig. 2, there were many reasons why students did not go to dental institutions. The most common reasons were “the children have no oral problems” and “time factors”.

Five questions were used to assess oral health knowledge and attitudes. The results are shown in Table 5. The correct answers for the “pit and fissure sealants can prevent dental caries in adolescents” and “fluoride is useless for dental protection” questions were not known by 34.75% of the parents of visually impaired students and 51.79% of the parents of hearing impaired students. The rate of correct answers to the above two questions was higher for the teachers than for the parents.

**Table 5:** The percentage of response on questions of oral health knowledge and attitude

	Health knowledge and attitude	Visual impairment				Hearing impairment		
		Agree (%)	Disagree (%)	Unknown (%)		Agree (%)	Disagree (%)	Unknown (%)
**Parents**	1. There is no need to treat deciduous teeth caries	13(11.02%)	91(77.12%)	14(11.86%)		11(19.64%)	31(55.56%)	14(25.00%)
	2. Fluoride is useless to dental protection	45(38.14%)	24(20.34%)	49(41.52%)		25(44.64%)	7(12.50%)	24(42.86%)
	3.Pit and fissure sealant can prevent dental caries of children	73(61.86%)	4(3.39%)	41(34.75%)		21(37.50%)	6(10.71%)	29(51.79%)
	4.Teeth are born good or bad, no correlation with the protection	4(3.39%)	100(84.75%)	14(11.86%)		7(12.50%)	36(64.29%)	13(23.21%)
	5.It is important to do oral examination regularly	106(89.83%)	3(5.36%)	9(16.07%)		49(87.50%)	1(1.79%)	6(10.71%)
**Teachers**	1. There is no need to treat deciduous teeth caries	2(6.25%)	29(90.63%)	1(3.12%)		3(13.04%)	16(69.57%)	4(17.39%)
	2. Fluoride is useless to dental protection	29(90.63%)	1(3.13%)	2(6.25%)		19(82.61%)	0	4(17.39%)
	3.Pit and fissure sealant can prevent dental caries of children	28(87.50%)	3(9.37%)	1(3.13%)		19(82.61%)	0	4(17.39%)
	4.Teeth are born good or bad, no correlation with the protection	1(3.13%)	30(93.74%)	1(3.13%)		4(17.39%)	16(69.57%)	3(13.04%)
	5.It is important to do oral examination regularly	28(87.50%)	3(9.37%)	1(3.13%)		21(91.30%)	2(8.70%)	0

### Results of the multivariate logistic regression analysis

To determine the factors affecting dental caries, we analysed the relevant data, such as age, sex, parent’s education, and oral health-related knowledge and attitudes. For the multivariate logistic analysis, the score of not using fluoride was set as “0”, and the score of using fluoride once a year was set as “1”. A score of “1” was assigned as a low parental education level, a score of “2” was assigned as a medium parental education level and a score of “3” was assigned as a high parental education level. As shown in Table 6, the results of the multivariate logistic analysis showed that fluoride use and parents’ educational background had an impact on the caries experiences of the participating students (*P* < 0.05). The prevalence of dental caries among visually impaired students who did not use fluoride was 2.41 times higher than that among those who used fluoride twice a year, with a 95% confidence interval of 1.06–5.53. The prevalence of dental caries in the offspring of highly educated parents was lower than that of the other groups. The frequency of brushing teeth every day and parents’ educational background had an impact on the caries experiences of students with hearing impairment. The risk of dental caries among those who brushed their teeth less than once a day was 4.15 times higher than that among those who brushed their teeth twice a day, with a 95% confidence interval of 1.24–13.92. Parental educational level was also a risk factor for hearing impairment in students.

**Table 6:** Multiple logistic regression analysis for the dental caries status of students

	variable		B	S.E.	Waldχ2	df	*P*	OR	95% C.I.	
									L	U
Visual impairment	Fluoride use	Once/ year	0.88	0.42	4.36	1	0.037	2.42	1.06	5.53
	Parent’s education background	low (junior school or below)	2.17	0.64	11.46	1	0.001	8.75	2.49	30.69
		median (senior school)	1.93	0.62	9.75	1	0.002	6.86	2.05	23.00
Hearing impairment	Frequency of brushing/ day	< once / day	1.42	0.62	5.32	1	0.021	4.15	1.24	13.91
	Parent’s education background	median (senior school)	1.50	0.59	6.53	1	0.011	4.50	1.42	14.27

## Discussion

At present, the oral hygiene status of students with hearing and visual impairment still poses great challenges. In this study, a special group of hearing impaired and visually impaired students were selected to undergo an oral examination and an oral hygiene habits questionnaire survey. The prevalence of dental caries in visually impaired and hearing impaired students were 66.10% and 66.07%, with a mean DMFT of 2.71 ± 3.06 and 2.57 ± 2.83, respectively. We first compared the DMFT results with similar studies abroad. The DMFT results of visually impaired students in this study was higher than most of the similar studies abroad, such as Sudan (Khartoum State: 0.4 ± 0.7) and India (New Delhi: 2.08 ± 1.86; Bengaluru: 1.32 ± 1.36), Iran (Tehran: 0.81 ± 1.15), while it was similar to the study conducted in Saudi Arabia (Riyadh: 2.13 ± 2.63 ) [14–17]. For the hearing impaired schoolchildren, the DMFT results in our study was higher than India ( Bhimavaram: 1.9 ± 1.3) and lower than Thailand (Nakhon Pathom: 3.90 ± 3.22) [18, 19]. The above results suggested that local eating habits, government policies and oral health attention might vary in different countries which result in the difference of oral hygiene.

Although, the prevalence of dental caries and DMFT results were lower than those of our previous study of students with visual impairment (78.68%) in Northeast China in 2019 [20], which may be related to more and more oral health programs were conducted by Chinese government and more and more attention had been drawn to oral health status of hearing impaired and visually impaired schoolchildren. We further found the prevalence of dental caries, DMFT and the prevalence of first molar in this study was higher than our previous study among healthy 12-15-year-old school children in Liaoning Province in China [21] and healthy students of the Fourth National Oral Health Survey in China [13]. The above results indicated that hearing and visual impairment in schoolchildren may affect the learning and hands-on ability, which might result in the worse oral hygiene habits than normal people. Likewise, these results indicated that oral health services for students with hearing impairment and visual impairment are underutilized.

It was found the periodontal health of visually impaired students was not better than that in our previous studies from 2019, the prevalence of gingival bleeding increased from 44.46–52.08% [20]. It was precisely during the COVID-19 epidemic period that many oral health programs in schools and care homes were disrupted or suspended, which may directly affected the students’ oral health. The periodontal health status of hearing impaired students was better (gingival bleeding: 17.9%, calculus: 42.9%) than the visually impaired students. These results were consistent with other studies that visual impairment was shown to have a greater impact on oral hygiene [22]. The prevalence of gingival bleeding and calculus of visually impaired and hearing impaired students were lower compared to the results of healthy adolescents in Findings from the 4th National Oral Health Survey in 2018 (61.0% and 67.3% ), Beijing area in 2018 (61.0% and 67.3%) and Gansu province in 2023 (70.2% and 85.6%) in China [23, 24]. These results were inconsistent with the view that oral hygiene condition worsened with the increasing severity of visual impairment [22]. It maybe due to the small sample size of visual and hearing impaired students. We further found that the periodontal status of males was worse than that of females, which was consistent with other studies about periodontal status of normal students [25]. It might be due to females have better oral hygiene habits, so that plaque removal would be much cleaner.

We further found that fluoride use and daily brushing frequency were strongly associated with the prevalence of dental caries in visually impaired students and that daily brushing frequency was a risk factor for dental caries. Multivariate logistic regression analysis showed that fluoride had a protective effect on dental caries and the risk of dental caries in those using fluoride once a year was 2.41 times that of those using fluoride twice a year. However, the awareness of fluoride and pit and fissure sealants among both teachers and parents is still low. It has been proposed by The American Dental Association that fluoride can effectively prevent dental caries [26, 27] and fluoride and pit and fissure sealants should be used to prevent dental caries in advance [28]. Other studies have also found that at the end of 12 months there was a significant decrease in OHIS scores in the fluoridated group, and there was a reduction in *Streptococcus mutans* and *Lactobacillus counts* [29]. In hearing impaired students, the frequency of tooth brushing is strongly associated with dental caries, However, some students still cannot master the correct brushing method. Studies have suggested the use of good verbal guidance and tactile assistance for improving tooth brushing methods [30, 31]. Therefore, it may also be necessary for professionals to provide oral hygiene guidance and verbal instructions to fully explain simple information.

We found that parental education level was a risk factor for dental caries in both visually impaired and hearing impaired students. Other studies have shown similar results: teacher- and parent-supervised toothbrushing with fluoride toothpaste can be safely targeted to hearing impaired children and can enable a significant reduction in plaque and gingival scores [32]. For visually impaired students and hearing impaired students, family education is the primary way for students to understand external knowledge. In contrast, they have relatively little access to obtain oral health knowledge through public channels. Therefore, relevant departments should strengthen oral health education for these groups.

According to statistics from the Ministry of Education, there were 2,244 special education schools nationwide in 2020, with an increase of 538 from 1,706 schools in 2010 [33, 34], indicating that an increasing number of students with special needs are receiving education through special education schools. Economic factors, traffic factors, and inconveniences in medical treatment are the main reasons why they do not seek timely medical attention. At present, attention from society and corresponding public health guarantees are lacking for this population [35]. Therefore, the government should provide oral health care for special populations based on the above points.

This study focused on the oral health status of visually or hearing impaired students in northeast China. Compared to the oral health status of healthy students, fewer studies have been conducted on visually or hearing impaired students, which has important implications for policy-making. Moreover, the questionnaire included teachers’ awareness of oral health knowledge and attitudes. Most of the students in this study lived in school, therefore, teachers’ awareness of responses to questions on oral health knowledge and attitudes had a great impact on oral health status. However, this study also has limitations. A cohort study was not conducted in this study because of time and economic costs. Further cohort studies should be conducted in these populations for long-term follow-up and investigation. The incidence of traumatic dental injuries and malocclusion was not included. Due to the strict admission system of special education schools, although there is no complete random sampling, this study adopted census of two independent special education schools. The samples included students from both urban and rural areas, and the ratio of males to females was similar, which basically reflects the characteristics of the population of disabled students. Follow-up work will be promoted as much as possible, such as the expansion of the sample size in the future. Likewise, we should add caries activity detection as an indicator of caries risk in follow-up investigations to help examiners understand students’ oral conditions more intuitively.

## Conclusions

The oral health situation of visually impaired and hearing impaired students remains severe. It is still necessary to expand the coverage of public oral health services for this special population and strengthen comprehensive interventions for oral diseases of students with special needs to promote the oral and general health of this population.

## Data Availability

The data that support the findings of this study are available from the corresponding author upon reasonable request.
